# Evidence for the Contribution of the Hemozoin Synthesis Pathway of the Murine *Plasmodium yoelii* to the Resistance to Artemisinin-Related Drugs

**DOI:** 10.1371/journal.pone.0032620

**Published:** 2012-03-05

**Authors:** Benoit Witkowski, Joel Lelièvre, Marie-Laure Nicolau-Travers, Xavier Iriart, Patrice Njomnang Soh, Fatima Bousejra-ElGarah, Bernard Meunier, Antoine Berry, Françoise Benoit-Vical

**Affiliations:** 1 CNRS, LCC (Laboratoire de Chimie de Coordination), and Université de Toulouse Paul Sabatier, UPS, INPT, LCC, Toulouse, France; 2 Service de Parasitologie-Mycologie, Centre Hospitalier Universitaire de Toulouse, and Faculté de Médecine de Rangueil, Université de Toulouse Paul Sabatier, Toulouse, France; 3 UMR 152 IRD-UPS, Université Toulouse III Paul Sabatier, Toulouse, France; 4 Palumed, Castanet-Tolosan, France; Barcelona Centre for International Health Research/Hospital Clinic/IDIBAPS/University of Barcelona, Spain

## Abstract

*Plasmodium falciparum* malaria is a major global health problem, causing approximately 780,000 deaths each year. In response to the spreading of *P. falciparum* drug resistance, WHO recommended in 2001 to use artemisinin derivatives in combination with a partner drug (called ACT) as first-line treatment for uncomplicated falciparum malaria, and most malaria-endemic countries have since changed their treatment policies accordingly. Currently, ACT are often the last treatments that can effectively and rapidly cure *P. falciparum* infections permitting to significantly decrease the mortality and the morbidity due to malaria. However, alarming signs of emerging resistance to artemisinin derivatives along the Thai-Cambodian border are of major concern. Through long-term *in vivo* pressures, we have been able to select a murine malaria model resistant to artemisinins. We demonstrated that the resistance of *Plasmodium* to artemisinin-based compounds depends on alterations of heme metabolism and on a loss of hemozoin formation linked to the down-expression of the recently identified Heme Detoxification Protein (HDP). These artemisinins resistant strains could be able to detoxify the free heme by an alternative catabolism pathway involving glutathione (GSH)-mediation. Finally, we confirmed that artemisinins act also like quinolines against *Plasmodium via* hemozoin production inhibition. The work proposed here described the mechanism of action of this class of molecules and the resistance to artemisinins of this model. These results should help both to reinforce the artemisinins activity and avoid emergence and spread of endoperoxides resistance by focusing in adequate drug partners design. Such considerations appear crucial in the current context of early artemisinin resistance in Asia.

## Introduction

Malaria is still the parasitic disease with the highest impact on public health in endemic areas with 781.000 deaths recorded in 2009 [1]. Even though the price of efficient medications and the lack of correct healthcare infrastructures are partially responsible for this alarming situation, the spread of drug-resistance parasites remains the major problem. While ACT (Artemisinin-based Combination Therapies) are now widely used and are often the only one recommended treatments against all *Plasmodium falciparum* strains, early-resistance foci in South-East Asia have appeared recently [2] and pose a threat to these current therapies.

To act against multi-drug resistant parasites, a detailed understanding of the mode action of the drugs is urgently needed. The rodent malaria models enabled the selection of parasites that are resistant to different antimalarial drugs, including recent molecules. In this context, a long-term drug pressure was carried out on *P. yoelii nigeriensis* in parallel with two main molecules: chloroquine (CQ) and artemisinin (ART). The goal of this project was to set up a malaria parasites model, in order to provide informations about the mechanism of action of artemisinin and its derivatives [3].

## Results and Discussion

### Drug pressures conferred multi-resistance to lysosomotropes (*i.e.* drugs targeting the parasite food vacuole)

**Figure 1:**
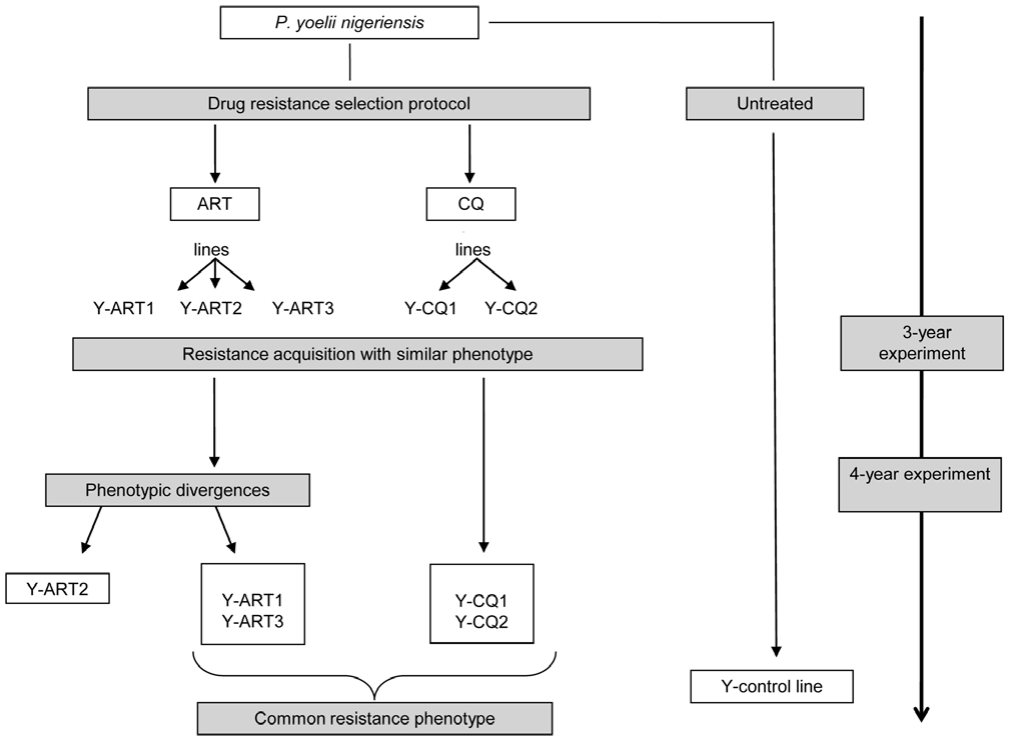
Experimental scheme of the 5-year drug resistance selection protocol.

The selection of drug resistant lines was carried out over 5 years by submitting in parallel the parasite strain *P. yoelii nigeriensis* to progressively increased doses of ART and CQ (Figure 1). After 3-year of drug pressure, the Fink's test [4] was used to evaluate the response of these selected parasite lines to the different drugs used for the selection pressures. Experiments were carried out on one strain of each line randomly chosen (Y-ART3 and Y-CQ2) and on the Y-control line (data not shown). Mice infected with any of the selected resistant lines could not be cured by treatments with ART (120 mg/kg sub-cutaneous (s.c.) whereas parasites of the Y-control line remained sensitive to these drug regimen since no parasite could be detected up to 30-days in the mice infected by this strain. High doses of CQ did not cure mice infected by selected resistant lines as well. On the other hand, 2 out of 3 of the mice infected with the Y-control line and treated with CQ (185 mg/kg s.c.) were cured. Even if a unique dose of CQ (185 mg/kg by s.c. injection) did not totally cure the mice infected by the Y-control line, CQ treatment was much more effective against the Y-control parasite growth than it was against the selected lines. The Fink's test was therefore not adapted to study CQ resistance as the maximal dose of CQ was too close to the dose that killed 50% of the mice (LD_50_ = 195 mg/kg s.c).

However, we can see here that whatever the drug initially used for selection (CQ or ART), all the resulting lines tested were resistant (or less sensitive) to these two molecules indicating a multi-drug resistance phenotype.

To quantify resistance acquisition *ex vivo* short-term cultures of *P. yoelii* were therefore performed. This assay was done on the resistant-selected lines and on the Y-control line (Tables 1, 2). The results showed that there was a considerable decrease in the susceptibility of all these selected lines, to quinolines (chloroquine, mefloquine and quinine) and to endoperoxides (artemisinin, artesunate, artemether, and artemisone [5]). As previously observed with the Fink's test, a phenotype of multi-drug resistance was obtained after resistance selection done with CQ, or ART. Whatever the molecules used for selection, each line exhibited a phenotype with a high resistance index (RI: corresponds for a given drug to the ratio of the IC_50_ value of the resistant line over the IC_50_ value of the control line) for quinolines with values, according to the strains tested, ranging from 67 to 152-fold for chloroquine, 68 to 120-fold for mefloquine and 17 to 39-fold for quinine (Table 1). Resistance to endoperoxides was also observed as well (Table 2), ranging according to the strains tested from 3 to 264-fold for artemisinin, 6 to 87-fold for artesunate, 14 to 53-fold for artemether and 13 to 21-fold for artemisone. However, this phenomenon was not observed with all drugs since the resistance index of sulfadoxine+pyrimethamine (SP) and atovaquone were closer to the control lines (Table 3) with RI ranged from 0.7 to 1-fold for SP and 0.3 to 2.2-fold for atovaquone.

**Table 1:** *Ex vivo* chemosusceptibility values for quinolines.

Lines	CHLOROQUINE				MEFLOQUINE				QUININE			
	pressure		release		pressure		release		pressure		release	
	IC_50_	RI	IC_50_	RI	IC_50_	RI	IC_50_	RI	IC_50_	RI	IC_50_	RI
Y-control	0.11±0.014	1	-	-	0.1±0.03	1	-	-	1.02±0.3	1	-	-
Y-ART1	12±3.3^*^	109	nd	-	10.3±1.7^*^	103	nd	-	17.6±2.5^*^	17	nd	-
Y-ART2	16.6±4.8^*^	150	nd	-	7.6±2.3^*^	76	nd	-	nd	-	nd	-
Y-ART3	9.5±1.8^*^	86	0.09±0.02	0.8	11.1±2.6^*^	111	0.03±0.004	0.3	24.3±5.5^*^	24	0.36±0.03	0.35
Y-CQ1	14±2.4^*^	127	0.073±0.03	0.66	12±2.9^*^	120	0.025±0.002	0.25	39±11^*^	39	0.51±0.05	0.5
Y-CQ2	16.8±1.8^*^	152	nd	-	7.4±3.3^*^	74	nd	-	27.7±17^*^	27	nd	-

**Table 2:** *Ex vivo* chemosusceptibility values for endoperoxides.

Lines	ARTEMISININ				ARTESUNATE				ARTEMETHER		ARTEMISONE	
	pressure		release		pressure		release		pressure		pressure	
	IC_50_	RI	IC_50_	RI	IC_50_	RI	IC_50_	RI	IC_50_	RI	IC_50_ (nM)	RI
Y-control	0.17±0.04^Ψ^	1	-	-	0.04±0.002	1	-	-	0.03±0.006	1	0.02±0.002	1
Y-ART1	1.8±0.84^*Ψ^	11	nd	-	0.43±0.04^*^	11	nd	-	0.6±0.075*	18	0.37±0.025*	19
Y-ART2	45±11.3^*^	264	nd	-	3.47±2.06^*^	87	nd	-	1.6±0.53*	53	0.57±0.12^#^	29
Y-ART3	0.8±0.11^*Ψ^	5	0.06 ± 0.009	0.4	0.38±0.01^*^	10	0.02±0.003	0.3	0.4±0.06*	14	0.28±0.04*	14
Y-CQ1	1.36±0.03^*Ψ^	8	0.05 ± 0	0.3	0.51±0.23^*^	13	0.02±0.003	0.3	0.6±0.008*	18	0.42±0.025*	21
Y-CQ2	0.9±0.2^*Ψ^	5	nd	-	0.40±0.10^*Ψ^	10	nd	-	0.5±0.033*	15	0.31±0.037*	16

**Table 3:** *Ex vivo* chemosusceptibility values for not-lysosomotropes.

Lines	SULFADOXINE-PYRIMETHAMINE				ATOVAQUONE	
	pressure		release		pressure	
	IC_50_†	RI	IC_50_†	RI	IC_50_ (nM)	RI
Y-control	6.5±1.16	1	nd	-	32.2±5.7	1
Y-ART1	4.50^#^	0.7	nd	-	23.9±14.3*	0.7
Y-ART2	5.1±0.73	0.8	nd	-	70.1±19	2.2
Y-ART3	5.9±1.23	0.9	5.5±0.74	0.84	23.6±4.9*	0.7
Y-CQ1	5.0±0.9	0.8	4.5±0.13	0.7	18.6±3.2*	0.6
Y-CQ2	5.0^???^	0.8	nd	-	20.5±9.5*	0.6

Homogeneity of the RI of the different drugs was evaluated for all the selected lines. The results were homogeneous for 4 of the 5 selected lines. For these 4 lines, there was no statistical difference between the IC_50_ values (concentration of drug that inhibited 50% of parasite growth) for all the tested lysosomotropic drugs. Nevertheless, the drug selection protocol led, after a 4-year drug pressure, to the expression of different phenotypes in term of IC_50_ values for the line Y-ART2 (Figure 1). The Y-ART2 line has acquired a notably higher resistance to endoperoxide and especially to ART (resistance index up to 260) and its derivatives artesunate and artemether (Table 2), and has also acquired an unexpected *in vitro* resistance to atovaquone (RI = 2.2) (Table 3). Because of its divergent phenotype, this line was not included in the determination of the resistance phenomenon presumed to be common to the 4 other lines and will be discussed later in detail.

Excluding Y-ART2, the IC_50_ values for quinolines and for the endoperoxides of the 4 resistant lines were statistically higher than those of the Y-control line (p<0.05). For SP and atovaquone, no statistical difference was found between the IC_50_ values from the 4 resistant selected lines and the IC_50_ values from the control line. (SP p = 0.56, ATQ p = 0.15).

These homogeneous values pointed to a common factor in the acquisition of resistance to quinolines and endoperoxides in all these resistant-lines.

Moreover, the multi-resistance obtained in these 4 selected lines was restricted to lysosomotropic antimalarial drugs. A common mode of resistance, possibly localized in the food vacuole, could explain the behavior pattern shared by all these lysosomotropic drugs, whatever the molecule used to select the resistance.

After 5 years of continuous drug pressure, certain selected lines (experiments performed only for lines Y-ART3 andY-CQ1 randomly chosen) were maintained *in vivo* but without any drugs (release of drug pressure) over 20 passages. The resistance to lysosomotropes (quinolines and artemisinins) of these released lines was totally lost with IC_50_ values statistically not different from those of the Y-control line (p>0.05), whereas the sensitivity to SP remained unchanged (Tables 1, 2, 3). These data indicated that the resistance acquired in these conditions was unstable and easily lost in the absence of drug pressure, possibly by restoration to a Y-control-like phenotype, meaning there was a high biological cost for these drug-resistance parasite phenotypes.

**Figure 2:**
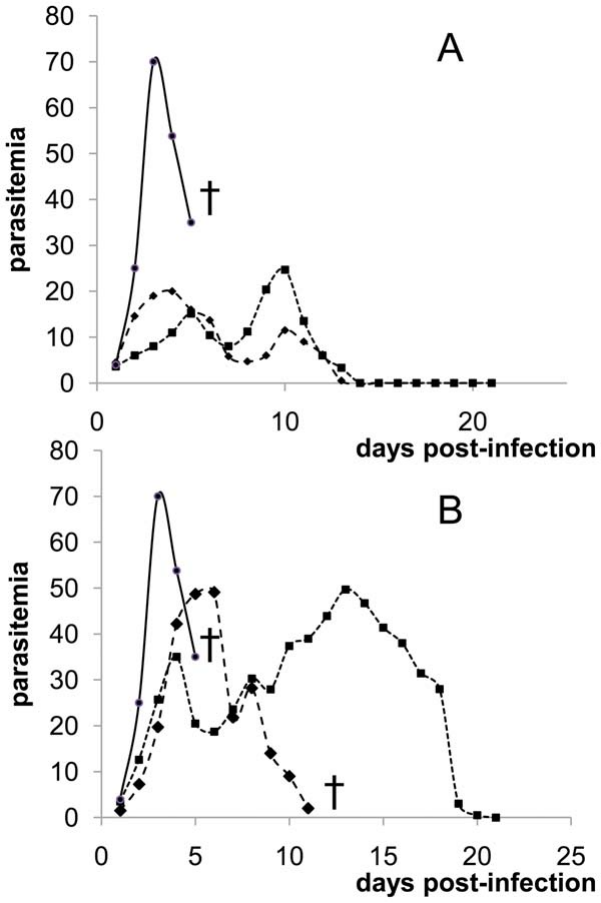
Parasitemia (%) profile of Y-control line, selected resistant lines and release lines (inoculum 1×10^8^ parasites). Drug resistance selection led to a loss of fitness and virulence as all the selected lines became not-lethal (A). Release of drug pressure partially restores fitness and virulence; lines could become lethal again (B). Parasitemia kinetic of the strains Y-ART3 (large square), Y-CQ2 (small square), Y-control (circle) and death of mice (cross) are shown. Figure 2 is one representative experiment from more than 5 experiments done with each of the strains tested.

*P. yoelii nigeriensis* is a virulent parasite able to kill mice within 5–8 days with a peak of parasitemia of 50–70% when using an inoculum of 1×10^8^ infected red blood cells. With the same inoculum, infections with selected parasites from groups Y-ART, Y-CQ did not kill mice and the levels of parasitemia were lower. Moreover, mice infected with these selected lines that are much less virulent were able to overcome the infection and cure themselves (Figure 2A). These results demonstrated that the acquisition of resistance certainly linked to a high fitness cost led to a loss of virulence of the parasite. Additionally, the release of drug pressure resulted in an enhancement of parasite virulence for each line (Figure 2B). These data exposed the relationship between virulence and resistance [6]. In summary, a homogeneous phenotype of multi-drug resistance was found after drug selection with ART and CQ for 4 out of 5 lines. This resistance was pleiotropic but restricted to lysosomotropic drugs.

### Resistance to lysosomotropic drugs was not associated to known resistance markers

**Figure 3:**
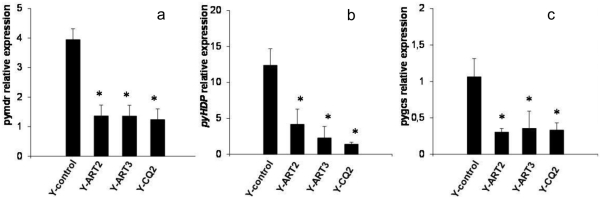
mRNA expression of *pymdr* (A) *pyhdp* (B) and *pygcs* (C). The results show relative expression in arbitrary units normalized to *pysts* expression (mean ± SEM of 3 independent experiments). *Comparison of Y-ART2, Y-ART3 and Y-CQ2 *vs* Y-control: p<0.03 by a one-way analysis of variance test and then p<0.05 by a pair-wise multiple comparison test *vs* Y-control group (Dunnett's method).

Multidrug resistance in *Plasmodium* is mainly associated to modifications of genes coding for proteins such as PfMDR1 orthologues [7] [8]. In rodent malaria models, amplification of the *mdr1* gene has been linked to CQ, ART and MQ resistance [9] [10]. However, comparative studies between our resistant lines and the control showed neither variations in the copy number of *pymdr1* (PY00245, an orthologue of *pfmdr1*) nor coding sequence differences that might explain the resistance. Moreover, mRNA quantification revealed statistically lowered transcriptional levels of *pymdr* linked with the acquisition of resistance (p = 0.002) (Figure 3A). This result might be mediated by transcriptional regulators. This low transcription could be linked to the ATP cost of ABC transporters such as *pymdr1* and the need for resistant parasites to limit useless energy consumption.

As a confirmation, penfluridol, a potent mdr1 inhibitor [11], did not reverse the resistance to mefloquine (neither CQ or ARTresistance; data not shown), whereas this inhibitor had been shown to reverse resistance in mefloquine-resistant lines of *P. yoelii* and *P. falciparum* [12] [11]. These data confirmed that the gene *mdr1* was not involved in our *P. yoelii* model of multi-drug resistance.

The major characteristic of these selected lines was the high resistance index to CQ (Table 1). In *P. falciparum*, resistance to CQ has been linked to mutations in the gene coding for the PfCRT channel, the chloroquine resistance transporter [13] [14]. However, no single-nucleotide polymorphism (SNP) was found here on the *P. yoelii* orthologue *cg10* of our selected lines (data not shown). Moreover, verapamil which reverts CRT-mediated CQ resistance [15], did not have any effects on CQ sensitivity in our resistant lines (data not shown). Additionally, chlorpromazine which is known to potently chemosensitize CQ resistant strains of *P. falciparum* to CQ [16], did not affect the CQ sensitivity of our resistant murine malaria models (data not shown) as observed by others [17].

Resistance of the selected lines to ART and to its derivatives was observed. ART resistance has been correlated to an increased mRNA transcription and thus increased expression of the protein TCTP (Translationally Controlled Tumor Protein), an identified target of artemisinin [18]. However, neither significant difference in mRNA expression nor SNP were detected in the present case in the Y-ART lines (data not shown), strongly suggesting a different resistance mechanism. These results obtained with a murine model should not be considered as directly transposable to the understanding of the resistance of the human malaria parasite, *P. falciparum*, but as a contribution on drug-resistance of malaria strains.

In summary, all these data suggested that these resistant lines circumvented the effects of lysosomotropic drugs by a global mechanism of resistance but without implicating the already known resistance factors. As a marked resistance to quinolines was ubiquitously observed in these selected lines, and as these drugs are known to act by interfering with hemoglobin catabolism, our investigations then focused on possible alterations in heme metabolism.

### Acquisition of lysosomotropic drug resistance was linked to impaired hemozoin production

Microscopically, lower hemozoin (Hz) concentrations were observed in the resistant parasites than in the Y-control. This finding was confirmed by the impossibility to concentrate late parasite forms from the selected lines with magnetic columns [19] contrary to the Y-control line, since this methodology acts trough the Hz paramagnetic properties.

**Figure 4:**
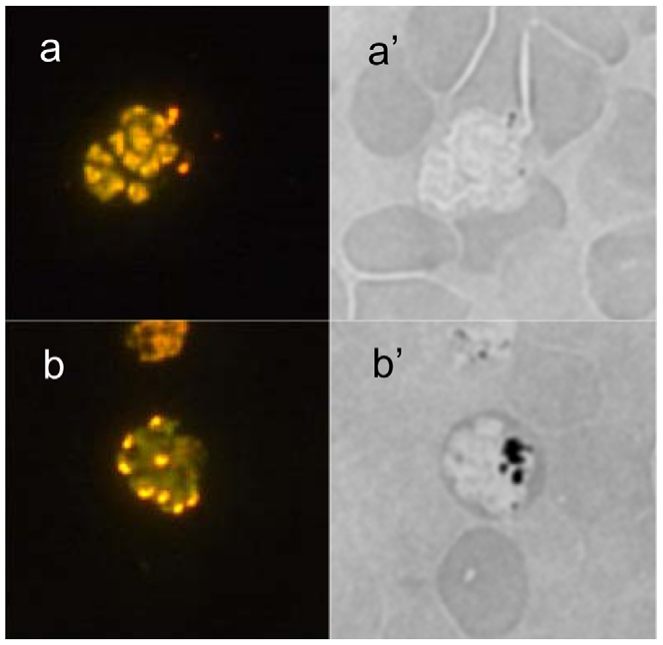
Epifluorescence microscopy. Y-ART3 line schizont stained with acridine orange (a) showed a typical multiple nuclei stage but no malaria pigment inclusion could be observed in a bright field (a'). Y-control line schizont stained with acridine orange (b) showed also a typical multiple nuclei stage but there is a large amount of malaria pigment also called hemozoin (the black inclusion) observed in a bright field (b').

**Figure 5:**
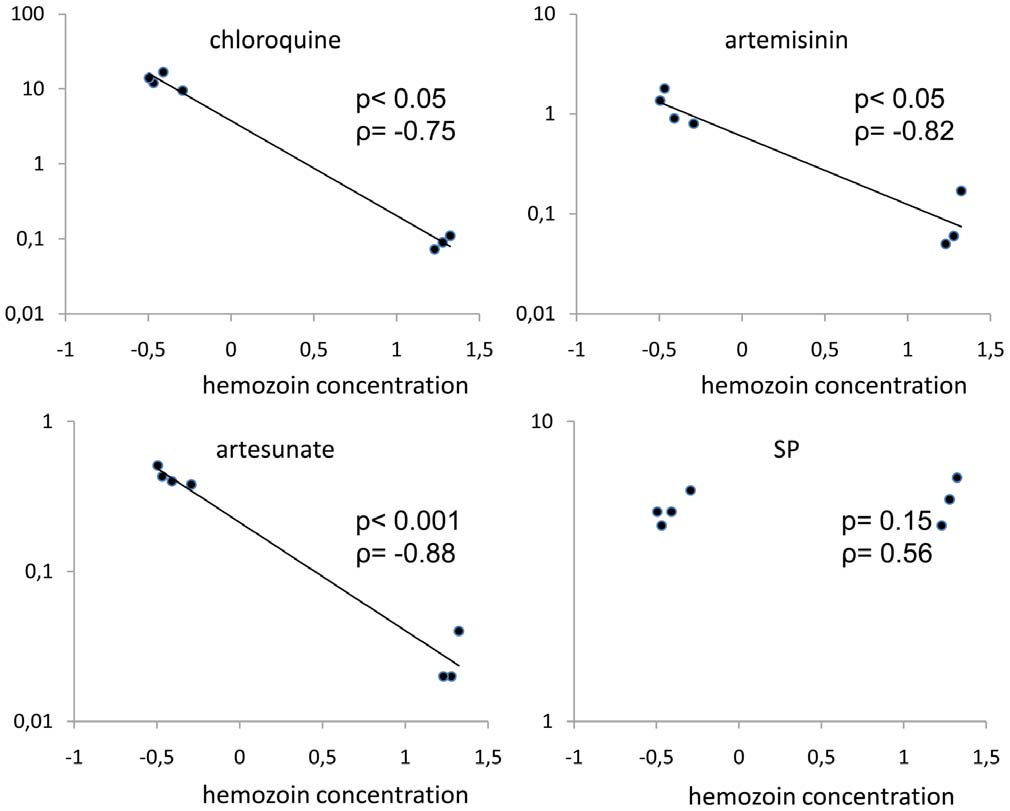
Correlation between IC_50_ values for CQ, ART, artesunate and SP in selected and control lines. The line for Y-ART2 was not included. IC_50_ values (mean of at least 3 independent experiments) are expressed in µM for CQ, ART, artesunate and as dilution (×10^6^) of SP stock solution (200 mg sulfadoxine+10 mg pirymethamine/mL). The Hz content is expressed as the log of concentration (pmoles of FPPIX/1×10^6^ trophozoites). A statistically significant negative correlation was found between the Hz content and the IC_50_ values for CQ (p = 0.03; ρ = −0.75) ART (p = 0.01; ρ = −0.82) and artesunate (p<0.001 ρ = −0.88) while there was no statistically significant correlation with the IC_50_ value for SP (p = 0.23, ρ = 0.7). *calculated by Spearman's rank-order correlation test.

The study of malaria pigment showed a significant reduction in the Hz content in the resistant lines compared with the control line (Table 4 & Figure 4). After the release of drug pressure and concomitant with the loss of resistance, the Hz content significantly increased, reaching values statistically not different from those of the Y-control line (p>0.05) (Table 4). A statistically significant inverse correlation was found between the Hz content and resistance to lysosomotropes (p<0.05 and ART ρ = −0.82, ATS ρ = −0.88, artemisone ρ = −1.00, artemether ρ = −0.98, Q ρ = −0.79, CQ ρ = −0.75, MQ ρ = −0.75). No correlation was found for not lysosomotrope drugs (SP : p = 0.23, ρ = 0.7 and ATQ: p = 0.233, ρ = 0.15) (Figure 5). These results clearly demonstrated the implication of the Hz synthesis pathway in the mode of action of the lysosomotropic drugs endoperoxide and quinoline.

**Table 4:** Hemozoin concentrations in Y-control and selected lines.

Lines	Hemozoin concentration	
	pressure	release
Y-control	21.08±3.82	-
Y-ART1	0.34±0.09*	nd
Y-ART2	0.40±0.24*	nd
Y-ART3	0.51±0.19*	19±3
Y-CQ1	0.32±0.08*	17±1
Y-CQ2	0.39±0.17*	nd

Hz formation was a common target for quinolines and endoperoxides and the disappearance of Hz would lead to a pleiotropic resistance phenotype as observed here. That may explain why the same phenotype was obtained between the lines resulting from the CQ or ART selection protocol. As expected, drugs known not to interfere with Hz-mediated heme catabolism such as SP and atovaquone did not show any reduced activity against the resistant lines. Since the decreased hemozoin concentration was the common parameter in all the resistant lines studied.

These data are in accordance with Klonis et al. recent results which indicate that potent artemisinin activity is dependent on hemoglobin digestion by the parasite with heme as target for endoperoxides [20]. However, hemoglobin digestion is crucial to parasite for amino-acids provision and such level of Hz decreased would indicate a quasi-arrest of hemoglobin degradation. Others demonstrated that hemoglobin digestion rate was not altered in a chloroquine- resistant *P. berghei* model, even if the heme rate was decreased in this murine malaria resistant strain in comparison with the sensitive one [21]. Decreased heme content as well as a decreased of Hz production observed add arguments to another pathway of heme degradation [21], [22].

We hypothesized that there was an alternative pathway involved in heme detoxification other than Hz formation. Therefore possible modifications in heme catabolism pathways were investigated.

### Resistant lines showed an alternative heme catabolism pathway

In malaria parasites, the majority of the toxic heme, resulting from hemoglobin digestion, is eliminated by crystallization to Hz. Recently, the protein HDP (Heme Detoxification Protein) was found to potently catalyze this biocrystallization process [23]. Among heme detoxification protagonist such as HRP2 (Histidine Rich Protein 2) and lipids, the HDP mediated one, has been described as being the most efficient and is ubiquitous to all *Plasmodium* species. Furthemore, *P. yoelii* HDP (PyHDP) was found to be as active as *P. falciparum* HDP in terms of Hz crystallization [23].

**Figure 6:**
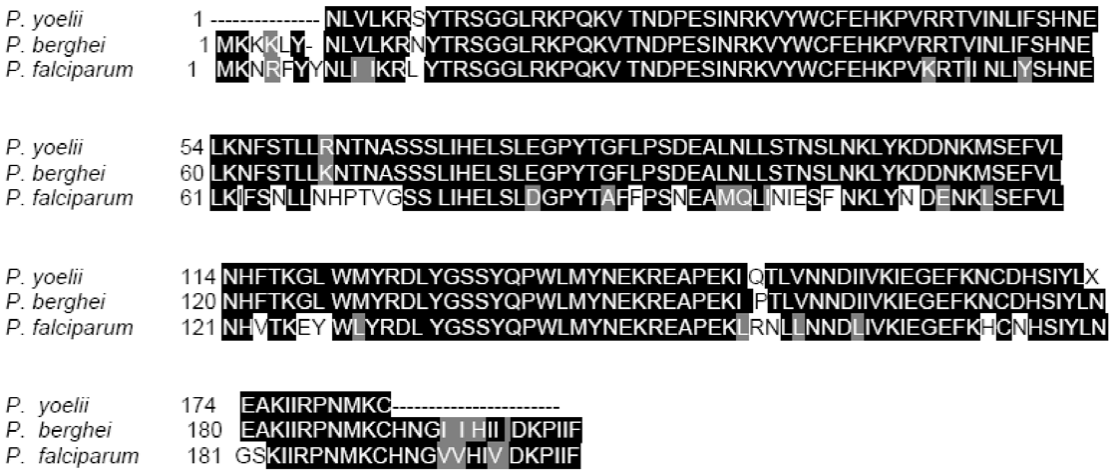
Alignment of predicted HDP sequence from the Y-control line cDNA sequence (*P. yoelii*) with *P. berghei* and *P. falciparum* referenced sequences (genes *pb000650.00.0* and *pf14_0446* respectively). The alignment was performed with ClustalW2 and treatment of the data with BoxShade 3.21; black and grey boxes indicate respectively identical and similar amino-acids between the 3 sequences studied. The Y-control sequence shares 97% identity with the *P. berghei* sequence and 77% with the *P. falciparum* sequence (data from ClustalW2).

**Figure 7:**
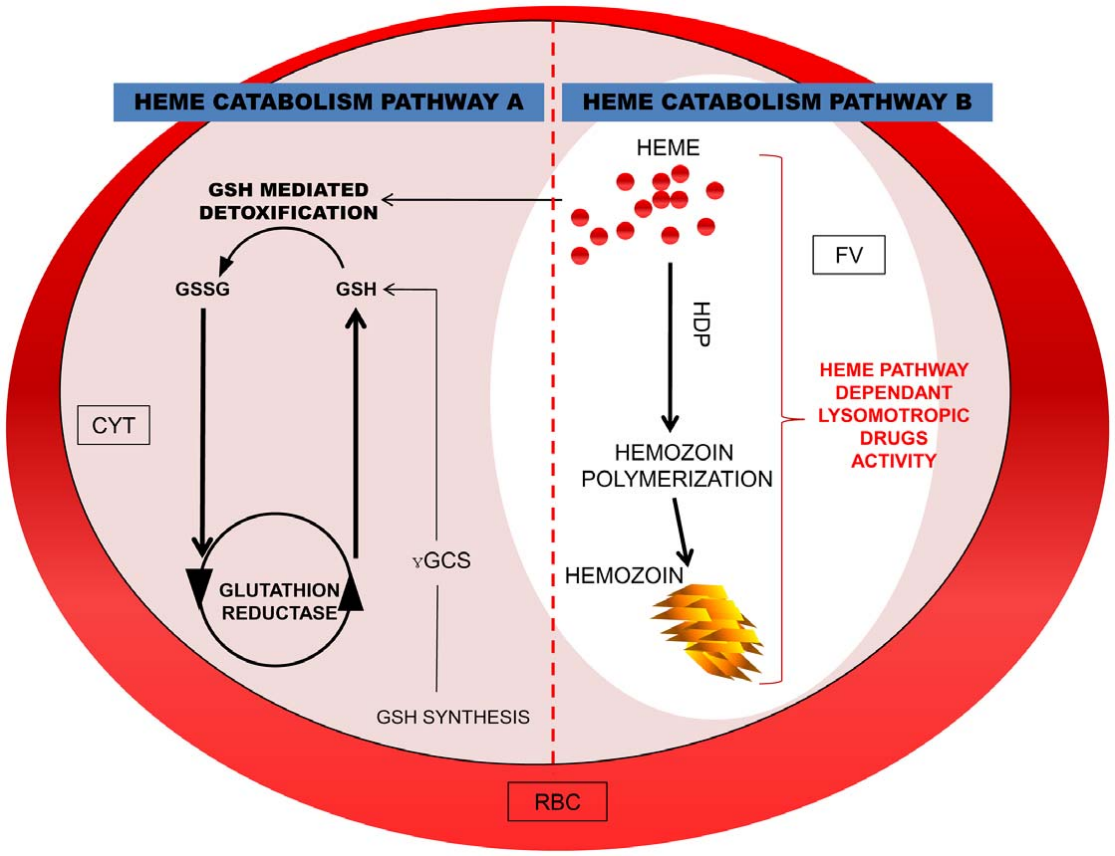
Scheme of Heme catabolim pathways in *Plasmodium*. Pathway A: GSH mediated heme catabolism. Pathway B: hemozoin biocrystallisation heme mediated catabolism. The pathway B is the standard way of heme catobolism, in majority present in sensitive and revertant lines instead of way A should be predominant in the resistance selected lines presented here. CYT : cytoplasm; FV: food vacuole; RBC: red blood cell.

As *pyhdp* is still not annotated in available databases (PlasmoDB, GeneBank), a partial sequencing of *pyhdp* was therefore carried out (Figure 6). No SNP were found to explain the lowered concentration of Hz in the selected lines. Nevertheless, mRNA quantification revealed that HDP was statistically under expressed in resistant lines compared with the Y-control line (p = 0.017) (Figure 3B) whereas the gene coding for HDP protein couldn't be deleted in *Plasmodium* highlighting the crucial presence of that gene for parasite viability [23]. This last finding corroborated the results obtained for the Hz concentrations in the resistant lines, thus confirming that HDP expression might be linked to lysosomotropic drug multi-resistance (Figure 7). This lower expression could be explained by the useless transcription of that gene upon the possible diminution of the Hz synthesis pathway. And thus the decrease in the Hz-based heme degradation pathway suggested the existence of a substitute pathway necessary to eliminate the toxic free heme (Figure 7). As a plausible alternative pathway was *via* glutathione-mediated heme detoxification [24], a comparison of the reduced glutathione (GSH) concentrations in the resistant lines *versus* the Y-control was carried out. The results showed that the concentrations of GSH were statistically higher in the resistant lines tested (p = 0.002) (Table 5). As expected, the GSH concentrations fell dramatically after the release of drug pressure to reach the level observed in the Y-control line (Table 5).

**Table 5:** GSH concentrations in selected, released and control lines.

Lines	GSH (nmoles/1×10^9^ parasites ± SEM)	
	pressure	release
Y-control	31.5±4	-
Y-ART2	136.5±13*	nd
Y-ART3	143.5±13*	26 ± 6
Y-CQ1	124±33*	22^#^

To validate this finding, chemosusceptibility to methylene blue (MB) was assessed on resistant and control lines. Methylene blue inhibits glutathione reductase and thus impedes the recycling of glutathione disulfide (GSSG) to reform GSH [25]: this depletion of GSH is the basis of its antiplasmodial mode of action.

The *in vitro* chemosensitivity assay to MB showed a statistically decreased effect of this compound on the resistant lines (p<0.003). This *in vitro* resistance to MB was lost after release of drug pressure, which reinforced the results of the GSH measurement (Table 6). A possible explanation could be that the increased concentration of GSH in resistant lines renders the GSH depletion effect of MB (and thus its antimalarial activity) less marked.

**Table 6:** *In vitro* chemosusceptibility of lines Y-ART3, Y-CQ1 and Y-control to methylene blue (MB).

Lines	MB IC_50_ (ng/mL ± SEM)†	
	pressure	release
Y-control	16.5±3.4	-
Y-ART3	850±414*	9.9±6.4
Y-CQ1	160±208*	12.4±8.3

We can suppose that the combination results published by others could be due to inhibition of GR on whole cycle (explaining the synergistic effects with ART) whereas inhibition of HZ pathway is occurred only on mature forms (explaining the antagonistic effects with some quinolines). Indeed, we have previously demonstrated the different moments of action on the erythrocytic malaria cycle of CQ and ART [26] that act from the mature trophozoite and since ring forms, respectively. However, on the present work, no combination was tested, we cannot thus speculate about the MB activity according to the partner drug and conclude about the ambiguous results obtained by others.

From this it may be deduced that there is a modification of the parasite GSH metabolic pathway in the resistant lines. The GSH level is known to be increased by over-expression of the limiting enzyme γ-glutamyl-cysteine-synthetase (γgcs) [27]. Surprisingly, we demonstrated that the transcription of *γgcs* was statistically lower in resistant selected lines (p<0.05) (Figure 3C). This surprising result could be related to a negative feedback existing between *γgcs* and heightened GSH concentration. . Considering the essential role of GSH for parasite development it might be possible that the present resistant lines used an alternative pathway to provide GSH. In a previous study, the provision of GSH *via* the host cells has been proposed [22].

In summary, different multidrug-resistant lines of *P. yoelii* were obtained. Whatever the molecules used for the selection pressures, the same alterations in chemosusceptibility were observed. This drug resistance phenotype seemed to be linked both to the metabolism of heme and of GSH.

### Possible modes of action of endoperoxides

The mode of action of ART and related compounds has been widely debated [28] [29]. Some studies indicated that the action of ART was mediated by heme-activation which generates toxic alkylating radicals after the opening of the endoperoxide bridge [30], and by inhibition of Hz production. ART could thus act by alkylating heme [31], some proteins [32] or by inhibiting Hz formation with adducts of ART-heme [33] [34], [35]. Other authors have suggested that heme was not a prerequisite for ART activity and suggested a not-heme mediated activation [36].

The results presented here suggest that these two opposing opinions could both be plausible if considered together. The quinolines are exclusively dependant on Hz production to be toxic and the resistance indices found in the present study for these drugs gave the highest values. On the contrary, the resistance indices of endoperoxides were more moderated. The divergence in resistance to these two different classes of drugs could be due to the mode of action of artemisinin-based drugs that involves multiple parasitic targets. Such a hypothesis was reinforced by the data obtained with the strain highly ART-resistant Y-ART2 (Table 2). This line showed a similar pattern of Hz concentrations as the two other ART-selected lines (Table 4). On the contrary, the chemosusceptibility data indicated that this line had very high resistance to ART which was statistically higher than the other selected lines (p<0.001) (the ART resistance index was about 260-fold compared with the control line). Additionally, the resistance of this Y-ART2 line was considerably increased for all the ART derivatives but the resistance to quinolines was not significantly increased (Table 1). Surprisingly, the sensitivity to atovaquone, a mitochondrial acting antiplasmodial drug, was statistically lowered in this line (RI of 2.2-fold) (p<0.05) while the sensitivity to SP was unchanged (Table 3). Previous studies have evoked the role of mitochondria in ART activity [37]. The unexpected correlation found between high resistance to ART and decreased atovaquone sensitivity could be explained by modifications in certain mitochondrial targets such as NADH oxidase or electron transport chain [38]. These latter data showed that this Y-ART2 line could have acquired a possible additional resistance pathway to ART different from the Hz-mediated resistance. However extended studies on this line remains necessary to elucidate the molecular mechanisms that drive this upper resistance to artemisinin.

In summary, resistance to ART and thus its mode of action seems to be not only linked to heme metabolism and further investigations are necessary to determine how this Y-ART2 line was able to resist such high concentrations of artemisininins.

Interestingly, artemisinin derivatives and synthetic endoperoxydes are also active against *Schistosoma* sp [39], [40], [41]. This parasite from the trematode class is obviously very different to *Plasmodium* but both parasites are able to produce hemozoin as a hemoglobin catabolism product. Quinolines, known to be dependant of the hemozoin pathway, were active on *schistosoma* as well [42] [43]. On the other way, artemisinins are weakly active (100 to 1000-fold less) against *Babesia* sp, a haematozoar close to *Plasmodium*, but which does not produce hemozoin trough its metabolism of hemoglobin [44]. Nevertheless, a weak activity of artemisinins exists on *Babesia* sp, probably arguing in favor of additional mechanism of action of these molecules beyond the heme catabolism. This hemozoin-independent activity, can also explain why the *P. yoelii* selected resistant lines here described are not totally resistant to artemisinins (same range as *Babesia* sp.) despite a quasi absence of hemozoin production. Finally, the “over-resistance” of the line Y-ART2 could be explained by such dichotomy of the mode of action of artemisinins and consequently, through the setup of additional mechanism(s) of resistance in this line. Furthermore, these data are supplementary arguments to plaid to the pivotal involvement of hemozoin metabolism pathway in the artemisinins activity but also to its not-exclusive contribution.

### Conclusion

In the present study, *P. yoelii* parasites that were multi-resistant to lysosomotropic drugs were selected. We demonstrated that this resistance is linked to major modifications in the parasites' heme metabolism. Even if this murine model certainly did not mimic resistance phenotype and resistance mechanism of human *P. falciparum* to tested drugs, it has permitted to provide new mechanistic elements of the artemisinin mode of action on a murine malaria parasite. Thus, we confirmed that quinolines act against *Plasmodium via* Hz production inhibition, but also that there is a link between the antiplasmodial action of endoperoxides and heme metabolism. However, we also suggested that endoperoxide activity is not only dependant on heme metabolism but can also act on other targets that could be mitochondria and certainly other targets. For field applications, this study also indicates that future treatment, currently in clinical trials [45], [46] combining an artemisinin derivative and a drug that interacts with alternative heme catabolism pathways (such as by reducing GSH content) could greatly enhance antiplasmodial activity and circumvent ART resistance. Such considerations appear crucial in the current context of early artemisinin resistance in Asia.

## Materials and Methods

### Parasites, mice and drugs

Swiss female mice (Janvier, France), 6–8 weeks old, were used to maintain parasites and select resistant lines. The *Plasmodium yoelii nigeriensis* strain was kindly provided by Dr. Irene Landau (Museum d'Histoire Naturelle, Paris, France). Chloroquine diphosphate (CQ), artemisinin (ART), mefloquine, quinine hydrochloride, verapamil, chlorpromazine and penfluridol were purchased from Sigma-Aldrich. Sulfadoxine-pyrimethamine (200 mg sulfadoxine plus 10 mg pyrimethamine per mL: Fansidar®) was purchased from Roche, France. Atovaquone and artemether were kindly provided respectively by Glaxo-Smith-Kline and Rhone-Poulenc. Artemisone wassynthesized according to previously published protocols [47] [48].

### Selection of drug resistant strains

Selections were carried out by submitting parasites to progressively increased doses of ART or CQ according to Peters and Robinson's protocol [49] with the following modifications. Female Swiss albino mice were infected intraperitoneally with 1×10^6^ infected red blood cells (IRBC). One hour later, drug was administered subcutaneously (s.c.) to animals. Follow-up of parasitaemia was daily monitored by caudal blood collection and giemsa-stained thin blood smears were read microscopically by at least two independent readers. When the parasitemia reached values ranging from 2 to 5%, blood was collected and injected into new mice and then drug pressure was reapplied corresponding to one passage. Selections of drug resistant parasites were performed over a period of five (5) years corresponding to about 340 passages for the selected lines and about 760 passages without any drug for the Y-control line. For artemisinin three mice were infected and treated and 2 mice were used for CQ. Two groups of selected lines corresponding to the drug CQ and ART used were then subdivided, studied in parallel and named Y-ART [1], [2], [3] and Y-CQ [1], [2] respectively (Figure 1).

The doses of the drugs ranged from 25 mg/kg to 185 mg/kg for CQ (vehicle: normal saline), 20 mg/kg to 120 mg/kg for ART (vehicle: DMSO) (vehicle: normal saline with 15% DMSO). The phenotype of resistance of these 5 selected lines (+ the control line) was firstly assessed after 3 years of drug pressure (by the Fink's test and the *ex vivo* test). Then the evolution of the phenotypes was monitored only with the *ex vivo* test. At the end of the 4^th^ year of drug pressures, phenotypic divergences appeared but remained stable during all over the 5^th^ year of the experiment. To explore stability or reversion of resistance, 5-year old selected strains were maintained in mice (as previously described) but without drug treatments for at least 20 passages.

### Evaluation of *in vivo* chemosusceptibility

The Fink's test was used to determine the effect of drugs *in vivo* [4]. Mice were infected with 1×10^7^ infected red blood cells (IRBC) from the selected or the control lines after 3-year old selected strains and treated with unique drug doses (ART 120 mg/kg, CQ 185 mg/kg; the controls were obtained by treating infected mice with only drug vehicle (DMSO, normal saline or normal saline with DMSO 15%). Parasitemia was monitored until 1 month after infection.

### *Ex vivo* chemosusceptibility assay

Heparinized 6–10% parasitemia infected blood was collected by retro-orbital puncture and was twice washed with RPMI. The red blood cell (RBC) pellets were resuspended in RPMI supplemented with 25% FCS and 0.5 µg/mL hypoxanthine and distributed in 96-well microplates prefilled with 100 µL of drug/well for a final volume of 200 µL and a final hematocrit of 2%. Immediately, ^3^[H] (0.05 mCi final) was added and the plates incubated at 37C°, 5% CO_2_ for 24 hours. The plates were then submitted to freeze-thaw cycles to lyse the cells and nucleic acids were collected on fiberglass filters. Tritium incorporation was measured with a microbeta counter (Perkin-Elmer).

To investigate the effect of chemosensitizers, parasites were co-incubated following the methodology used to determine *ex vivo* sensitivity (ART, CQ and mefloquine) with chemosensitizing agents (verapamil, penfluridol, and chlorpromazine purchased from Sigma-Aldrich) at concentrations corresponding to their IC_20–30_.

### Animal procedures

All procedures involving living animals were carried out in the animal facilities of the Parasitology Department of the Toulouse (France) University Hospital under the control of the National Veterinary Services and performed according to European regulations (EEC directive 86/609 dated 24 November 1986). The staff in charge of the animal experiments had received the appropriate training. All *in vivo* studies were approved by the French Institutional Animal Experimentation Ethic Committee (approvals MP/R/05/32/11/07 and MP/04/04/01/08 for the selection of drug resistant clones and the Fink's test, respectively).

### Determination of gene copy number

Genomic DNA was extracted from infected RBC (IRBC) with a High Pure PCR template preparation kit (Roche), in accordance with the manufacturer's instructions. The RT-PCR reaction was performed on a LightCycler V1.5 (Roche) in a 10 µL final reaction mixture with 2.5 µL DNA template, 0.5 mM primers (Eurogenetc, Belgium) (TUB1/2 and MDR1/2 (Table S1)), 4 mM MgCl_2_ and 1 µL Fast Start SYBR green (Roche). The gene amplification copy was estimated by comparison with the Cp value of *β-tubulin* (1 copy reference gene) and the Ct value of *pymdr* (target gene) between the sensitive control strain and the resistant strains through the 2^−ΔΔCt^ method [50]. Each reaction was performed in triplicate and the experiment repeated three times. Primers were designed using PRIMER3 software and the PLASMODB database.

### mRNA extraction and quantification

Parasites were synchronized with a percoll gradient to extract mRNA from homogeneous stages. Nine parts of percoll (Sigma) were mixed with one of 10X HBSS without Na_2_HCO_3_ (Sigma) and adjusted to pH 7 with 0.3 M hydrochloric acid. A discontinuous gradient was obtained by successive layering of decreasing concentrations of the percoll solution (80, 75, 72, 68, 60, 45 and 20%). Heparinized whole blood was then gently deposited on the gradient and it was centrifuged 15 min/1,900 g. Parasites were isolated according to their cycle age with older forms in the two first blood layers and younger forms in the third and fourth layers. The selected blood layers (late trophozoites and young schizonts) were collected and washed twice in cold Phosphate Buffer Saline (PBS). Blood pellets were then mixed with 800 µL of Trizol (Invitrogen) and stored at −80°C until RNA extraction. Total RNA was extracted by adding 200 µL of chloroform and spinning at 4°C 18,000 g/30 min. The supernatants were mixed with an equal volume of 70% ethanol and placed on an RNeasy MiniElute Cleanup column (Quiagen). The last part of the RNA extraction was performed in accordance with the supplier's protocol. RNA samples were stored at −80°C until reverse transcription. Before carrying out RT, gDNA was eliminated from the RNA by RQ1 DNase I treatment (Promega). The absence of gDNA contamination was assessed by performing a *β-tubulin* RT-PCR reaction on DNase treated RNA.

Reverse transcription was carried out on 1 µg RNA with a Reverse Transcription System kit (Promega). Quantification was done by RT-PCR using the previously described protocol to estimate the *pymdr* copy number. The gene PY03295 (seryl-tRNA-synthetase) was used as a housekeeping gene. All primer couples (Eurogenetec, Belgium) (Table S1) were tested with serial log dilutions of cDNA to ensure the high efficiency of the reactions (above 95%). Primers were designed using PRIMER3 software and the PLASMODB database (www.plasmodb.org).

### Hemozoin extraction and quantification

Hemozoin extraction and assay were done following the protocol of Orjih et al [51] with modifications. In order to normalize our results for the absolute number of parasites, for each sample the parasitemia was microscopically determined by at least 2 independent readers, the RBC numeration was also determined and the volume of the sample treated as well. The RBC pellet was lysed with PBS plus 0.08% saponin. Each pellet was then re-suspended in SDS solution (2.5% SDS in Tris-HCl 25 mM) and incubated overnight. The extracts were then centrifuged (1 h/18,000 g) and the Hz pellets harvested and washed with SDS solution. Hz decrystallisation was achieved by treatment for 2 h at room temperature with SDS solution plus 0.1 M NaOH and the Hz quantified by reading the absorbance at 405 nm. A reference curve was obtained with dilutions of FPIX chloride solution incubated in NaOH-SDS solution. The residual hemin/hemoglobin background level was estimated by treating the blood of healthy mice with the same protocol.

### Measurement of reduced GSH

Whole blood of infected mice (6 to 15% parasitemia) was collected (with heparin), diluted (1/3 v/v) with cold PBS and applied to a leukocyte removal disposable column (10 mL syringe filled with 100 mg of microcrystalline cellulose 20 µm (Sigma)). IRBC lysis was obtained by incubating 5 min on ice in 10 mL cold PBS containing 0.025% saponin. Parasites were washed twice with cold PBS and homogenized in three volumes of cold 5% sulfosalicylic acid. The suspension was submitted to three freeze/thaw cycles using liquid nitrogen and a 37°C water bath and spun at 4°C (10 min/18,000 g). 50 µL of supernatant was mixed with 150 µL of Tris-HCl/EDTA buffer pH 9 plus 10% of 5,5′-dithiobis nitrobenzene solution in DMSO. The absorbance (maximal peak at 412 nm) was determined at 405 nm using a 10 nm band pass. A reference curve was obtained by measuring the absorbance of serial GSH (Sigma) dilutions in 5% SSA.

### Epifluorescence microscopy

Infected blood from Y-control and Y-ART was collected (100 µL) with heparin and then washed with 1 mL PBS. 20 µL RBC were re-suspended in 1 mL acridine orange (Merck) solution (5 µg/mL in PBS) and incubated 5 min at room temperature. The blood was then washed 3-times with 1 mL PBS and smeared on a glass slide. Slides were observed immediately after preparation, with a Leica DPMI fluorescence microscope at the appropriate excitation wavelength (480 nm).

### Statistical Methods

To interpret and analyze the results, a logarithmic transformation was carried out to convert some not-Gaussian data to a normal distribution. Results were given as mean +/− SEM. To compare groups, a one-way or a Kruskal-Wallis one way analysis of variance tests were performed as appropriate and if a difference was found between groups, then a multiple comparison procedure (Dunnett's or Dunn's method) was done. The relationships between two variables were analyzed by Spearman's rank-order correlation test. A comparison was considered statistically significant if the p value was ≤0.05. All tests were performed using the SigmaStat (2.03) statistical program (SigmaStat, Heame Scientific Software, Chicago, USA).

## Supporting Information

Table S1 **Primers used for the RT-PCR and sequencing experiments.** NA = not annotated.
(DOC)
