# Supplementary material for: Evidence for the Contribution of the Hemozoin Synthesis Pathway of the Murine Plasmodium yoelii to the Resistance to Artemisinin-Related Drugs
Source: PLoS One. 2012 Mar 5;7(3):e32620. doi: 10.1371/journal.pone.0032620 (PMC3293827; doi:10.1371/journal.pone.0032620)
Supplement: Table S1 — Primers used for the RT-PCR and sequencing experiments. NA = not annotated. (DOC) [file pone.0032620.s001.doc]

| **primers** | **sequence** | **gene** |
| --- | --- | --- |
| GCS1 | 5’-CGT-CAC-TCT-TTG-TTC-GAG-ACC | PY01606 |
| GCS2 | 5’-TCT-TCG-CTC-CAC-ATT-TTT-GA |
| MDR1 | 5’-CAT-GCG-CCT-GGA-TTT-TTA-TC | PY00245 |
| MDR2 | 5’-GAT-TTT-TGC-AAT-CGT-GCT-CTT |
| STS1 | 5’-CCA-TAA-TGG-AGG-AAT-GTG-CAG | PY03295 |
| STS2 | 5’-ATG-CGC-ATA-ATG-GTT-GTT-CA |
| TCTP1 | 5’-AGA-TGG-AAT-GGG-AGC-TGA-TG | PY04896 |
| TCTP2 | 5’-AAG-GGT-TGT-GCC-TTT-GTT-TT |
| TUB1 | 5’-TAG-TGT-TCG-TGC-AGG-TCC-AT | PY05711 |
| TUB2 | 5’-TCG-CAT-CCT-TCT-GCT-TCT-TT |
| HDP1 | 5’-AAA-AGA-GAG-GCC-CCA-GAA-AA | NA |
| HDP2 | 5’-TTG-GGT-CTT-ATA-ATT-TTT-GCT-TCA |
| HDP-seqF | 5’-TTG-TAT-AAT-TTA-GTT-CTT-AAA-AGA-AG | NA |
| HDP-seqR | 5’-AAA-AAT-TAT-TGG-CTT-ATC-TAT-GA |
